# Operation for an Imperforate Anus

**Published:** 1826-07

**Authors:** 


### OPERATION FOR IMPERFORATE ANUS, AND TERMINATION OF THE RECTUM IN THE VAGINA.

In the January Number of Heckus, *Litherorische Annalen der gesammten Heilkunde*, for 1826, is a paper by Dr. DIEFFENBACK, a very intelligent and rising physician of Berlin, on Imperforate Anus: in which he recites a case where he performed the operation for the imperforate anus, complicated with cloaca with perfect success. The patient was a little girl about three months old, well grown for a child of her age, and appeared quite healthy. The external parts of generation were naturally formed, the anus was closed, and the fæces were discharged through a small opening about $\frac{1}{7}$ of an inch in diameter, in the upper and back part of the vagina. The operation for the removal of this deformity was made at two separate times. At the first, a curved director was introduced into the rectum through the opening in the vagina, and pressed downwards; a sharp-pointed bistoury was then introduced immediately behind the fossa navicularis, and carried towards the end of the director, but without cutting into it. This incision was extended near to the os coccygis, thus dividing the whole of the perinæum; and when the edges of the wound were separated, the extremity of the rectum could be seen terminating in a cul de sac. The lower part of the gut was then separated carefully with the bistoury from the posterior surface of the vagina, slit open, and allowed to lie in contact with the sides of the first-made wound. The parts were, after the operation, treated with a cold lotion, and when the suppuration begun, with a tepid fomentation. The opening from the rectum into the vagina, after having been once touched with lapis infunalis, was perfectly closed. For fourteen days, during which time the fæces passed through the newly made wound, nothing unfavourable occurred, and the edges of the wound at the end of that time cicatrized.

In three weeks after the first operation, the second was performed, namely, for the purpose of forming a new perinæum. Drs. Meyer and Gedike of Berlin, and Messrs. Coulson and Spry of London, were present. The operation was begun, by further separating the anterior surface of the rectum from the vagina, and the sides of the extremity of the rectum, being already adherent to the sides of the former incision; when this anterior part of the gut was separated, it was drawn backwards quite distinctly four or five times by the already adhering parts. The edges of the fore part of the old opening were now cut off, so that they might unite by the adhesive process when brought into contact. The deeper seated parts were drawn together by a suture, the ends of which were cut off quite close, and the integuments by two small pins, similar to those employed in hare-lip, over which the twisted suture was applied, and thus were the parts effectually secured in contact, and a new perinæum formed. Immediately after the operation, the persons present had the satisfaction of seeing the fæces escape through the artificial anus which remained. A small bougie was introduced into the rectum daily, and the greatest cleanliness directed to be observed. On the fifth day, the suture and needles were removed; a complete union had taken place, and thus was the object of the operation, namely, the formation of an artificial anus with the closure of the opening into the vagina satisfactorily accomplished.

